# Female Epispadias Presenting as Urinary Incontinence

**DOI:** 10.21699/ajcr.v8i2.548

**Published:** 2017-03-18

**Authors:** Asmir Jonuzi, Nusret Popovic, Zlatan Zvizdic, Emir Milišic, Kenan Karavdic, Dewan Paddy

**Affiliations:** 1 Clinic of Pediatric Surgery, Clinical Center University in Sarajevo, Bolnicka 25, 71 000 Sarajevo, Bosnia and Herzegovina; 2 Kind Cuts for Kids Australia.

**Keywords:** Epispadias, Bifid clitoris, Urethra, Urinary incontinence

## Abstract

Isolated female epispadias without bladder exstrophy is a rare congenital anomaly affecting 1 in 484,000 females. The presenting features of female epispadias are urinary incontinence and abnormal anatomical features. A 6-year-old girl presented with primary urinary incontinence who on physical examination had a bifid clitoris and labia minora. The vagina and hymen were normal. Voiding cystourethrogram showed no reflux and a funnel shaped proximal urethra. With the diagnosis of isolated female epispadias, one-stage reconstruction of the urethra, bladder neck, labia minora and clitoris was performed.

## CASE REPORT

A 6-year-old female child presented with urinary incontinence and abnormal appearing genitalia since birth. The patient’s family also reported a history of recurrent urinary tract infections. The child was a product of nonconsanguineous marriage, born through a normal vaginal delivery and had normal developmental milestones. Inspection of external genitalia revealed a bifid clitoris and depressed mons (Fig.1). The labia minora was under-developed and terminated anteriorly to the corresponding half of the bifid clitoris. The central urethra lying above the vagina, was short and widely open dorsally, communicating with an open bladder neck that was incontinent of urine. Although routine biochemical parameters were within normal ranges, urinalysis revealed more than 13 red blood cells and 7–8 white blood cells per high power field. Urine culture grew Proteus mirabilis. There were no abnormalities on detected on intravenous urogram and ultrasound. Voiding cystourethrogram showed a small bladder capacity (70-80 ml) bladder with no vesicoureteral reflux (Fig. 2). Pubic diastasis (width 2 cm) was noted.

Urethrocystoscopy showed a short, wide urethra, approximately 1 cm long, with splaying of the roof of the urethra and a wide bladder neck. The ureteric orifices were situated 2 cm from the bladder neck and were of normal caliber. With the diagnosis of female epispadias, a combined Young-Dees and urethroplasty procedure was performed. The salient features included urethral plate tubularization, bladder neck and the proximal urethra relocation into the intra-abdominal position. The continence procedure was done by creating posterior strip of mucosa 15 mm wide and 30 mm long extending distally from the midtrigone to posterior urethra. The procedure was completed with cosmetic reconstruction of the external genitalia. The pubic symphysis was approximated. Suprapubic and uretheral tube were placed. Suprapubic tube was removed on 21st postoperative day, and uretheral tube on 10th postoperative day.

**Figure:**
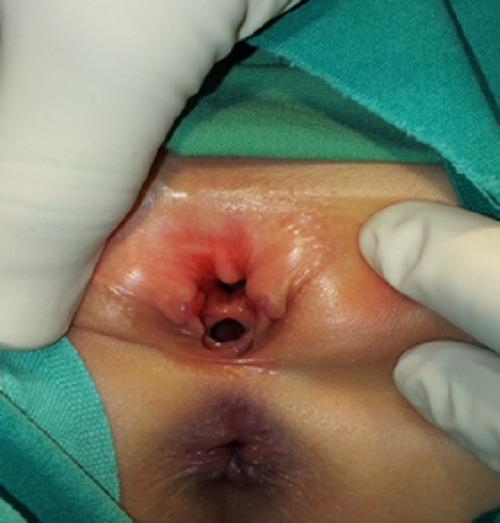
Figure 1: Bifid clitoris, depressed mons with deficient dorsal wall and normal vaginal opening

**Figure:**
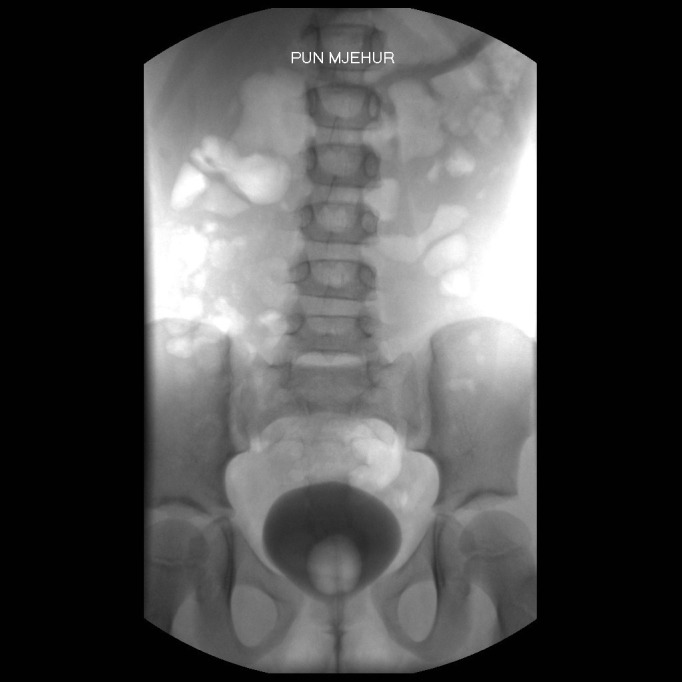
Figure 2: Micturating cystourethrogram before operation showing pubic diastasis and no vesico ureteral reflux.

At three month follow-up there was no incontinence of urine. Her mother reported that the child would express a sensation of a full bladder and a desire to micturate, with an ability to hold her urine and to initiate micturition voluntarily with complete bladder emptying. A postoperative voiding cystourethrogram after one year revealed a normal bladder neck and a lengthened urethra, with no reflux. A postoperative urodynamic study one year after the operation showed that the bladder capacity was 150 ml with a leak point pressure of 25 cm H2O. The postoperative cosmetic appearance was acceptable (Fig. 3).

**Figure:**
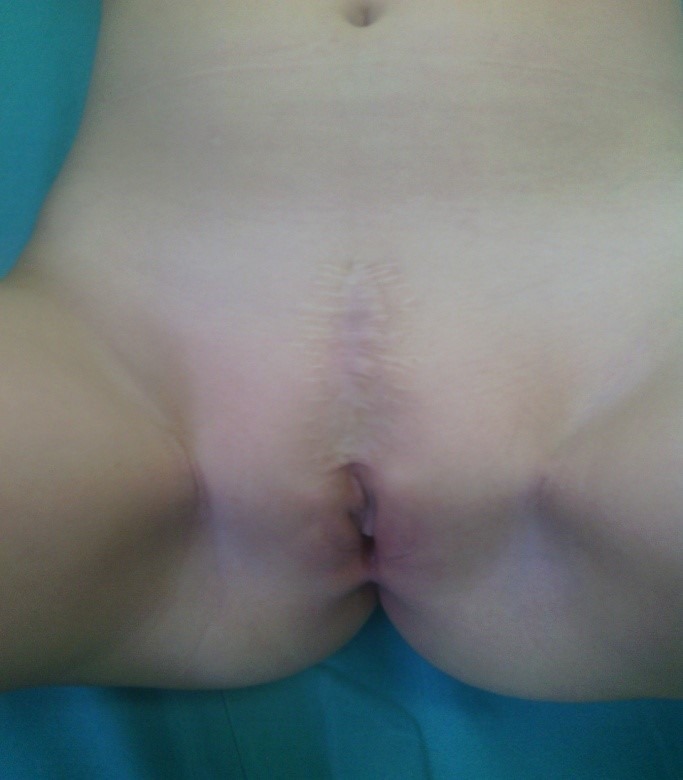
Figure 3: Postoperative appearance.

## DISCUSSION

Female epispadias is an uncommon congenital abnormality of the lower urogenital tract, which often presents with urinary incontinence, as in the index case. Usually it occurs sporadically but in some cases there is a strong genetic component.[1] The incontinence varies from continuous dribbling of urine without bladder filling to episodes of day-time stress-incontinence. Often the bladder capacity is reduced as a consequence of the lack of filling.[2] External genitalia can have varied appearance as classified by Davis ranging from lesser degrees with patulous urethral orifice to intermediate cases with urethra dorsally split along most of its length to the most severe cases which involve the entire length of urethra and bladder neck, rendering the sphincteric mechanism incompetent.[3] Milder forms of epispadias are extremely rare.[4] The objectives of surgical repair include achievement of urinary continence with preservation of the upper urinary tracts and the reconstruction of functional and cosmetically acceptable genitalia.[5] The same was achieved in the case presented. 

## Footnotes

**Source of Support:** Nil

**Conflict of Interest:** None declared

